# Evaluation of an Injectable Thermosensitive Hydrogel As Drug Delivery Implant for Ocular Glaucoma Surgery

**DOI:** 10.1371/journal.pone.0100632

**Published:** 2014-06-20

**Authors:** Lei Xi, Tao Wang, Feng Zhao, Qiongjuan Zheng, Xiaoning Li, Jing Luo, Ji Liu, Daping Quan, Jian Ge

**Affiliations:** 1 State Key Laboratory of Ophthalmology, Zhongshan Ophthalmic Center, Sun Yat-Sen University, Guangzhou, PR China; 2 DSAPM and PCFM Lab, School of Chemistry and Chemical Engineering, Sun Yat-sen University, Guangzhou, PR China; 3 Aier Eye Hospital Group, Changsha, Hunan, China; 4 Department of Ophthalmology, Second Xiang Ya Hospital, Central south university, Changsha, PR China; 5 Yale Eye Center, Yale University, New Haven, Connecticut, United States of America; Center for Molecular Biotechnology, Italy

## Abstract

In this study, a biodegradable thermo-sensitive hydrogel from poly(trimethylene carbonate)_15_-F127-poly(trimethylene carbonate)_15_ (PTMC_15_-F127-PTMC_15_) was designed and evaluated as an injectable implant during ocular glaucoma filtration surgery in vivo and in vitro. Mitomycin C (MMC) was loaded into this hydrogel for controlled released to prolong the efficacy and to reduce the long-term toxicity. The properties of the hydrogel were confirmed using ^1^H NMR and gel permeation chromatography (GPC). Compared to the Pluronic F127 hydrogel, the PTMC_15_-F127-PTMC_15_ hydrogel showed a good solution-gel transition temperature at 37°C, a lower work concentration of 5% w/v and a longer mass loss time of more than 2 weeks. The in vitro study showed that the drug could be released from PTMC_15_-F127-PTMC_15_ (5% w/v) hydrogel for up to 16 days with only 57% of drug released in the first day. Moreover, the cell toxicity, which was tested via LDH and ANNEXIN V/PI, decreased within 72 h in human tenon's fibroblast cells (HTFs). The in vivo behavior in a rabbit glaucoma filtration surgery model indicated that this hydrogel loaded with 0.1 mg/ml MMC led to a better functional bleb with a prolonged mean bleb survival time (25.5±2.9 days). The scar tissue formation, new collagen deposition and myofibroblast generation appeared to be reduced upon histological and immunohistochemistry examinations, with no obvious side effects and inflammatory reactions. The in vitro and in vivo results demonstrated that this novel hydrogel is a safe and effective drug delivery candidate in ocular glaucoma surgery.

## Introduction

Glaucoma filtration surgery [1], a classical surgery that creates a fistula to allow the aqueous humor to drain into the filtering bleb, has proven to be an effective method in the treatment of glaucoma, a leading cause of irreversible blindness worldwide [2]. However, 30%–50% of surgical procedures fail due to postoperative fibrosis related fistula block [3]–[5].

Mitomycin C (MMC) [6], [7], which is usually subconjunctivally used for 2–5 minutes with concentrations from 0.2–0.5 mg/ml [8], is the most effective and popular [9] antiproliferative drug for glaucoma filtration surgery especially in eyes at high risk for failure. However, MMC can only be used for a short period of time to avoid potential complications, including thinner blebs, hypotony and bleb leak [8], [10], [11], which are associated with uncontrolled application, making the application of MMC a double-edged sword.

Due to the long-term postoperative scar formation time, which lasts for over two weeks, a drug delivery vehicle that can maintain an effective drug concentration for an appropriate period to regulate postoperative proliferation and maintain bleb filtering with fewer complications is desirable.

Compared to many drug delivery vehicles, including liposomes [12], chitosan (CS) [13], collagen [14], cyclodextrin [15], pHEMA hydrogels [16] and polyester [17], the in situ formation of a thermo-sensitive hydrogel [18]–[26] stands out as an ocular topical drug delivery systems for its simple drug encapsulation and administration procedures. Pluronic F127is a well-known thermo-sensitive hydrogel and can exhibit a reversible solution (sol)-gelation (gel) transition in an aqueous solution at 25°C [27]. The FDA approved this hydrogel due to its enhancement of protein stability [28], lack of inherent myotoxicity [29] and excellent biocompatibility [30]. It has been extensively used for the treatment of burns [31], where it acts as a depot for the in situ controlled release of many drugs with subcutaneous injection [30], [32]–[38]. However, the relatively high critical gelling concentration (CGC) (16%) in aqueous solution and the fast degradation rate of a few hours have limited its application.

The modification of the two ends, especially hydrophobic modification, has been reported as a promising strategy to improve the stability of hydrogels derived from F127 [39], [40]–[42]. Because poly(trimethylene carbonate)(PTMC) shows excellent biocompatibility and absorbability, two oligomers of PTMC were chosen as the hydrophobic segments. The degradation of PTMC takes place via an enzymatic surface erosion process [43] and is less variable in vivo and free from the formation of acidic products that could induce an inflammatory response [44]. A thermo-sensitive hydrogel with a low CGC (3.5%) derived from a PTMC-F127-PTMC copolymer was subsequently obtained [39], [45].

In this study, a well-designed block copolymer PTMC_15_-F127-PTMC_15_ was synthesized, and the properties and releasing performance with MMC were assessed. The safety and efficacy of this delivery system in inhibiting the formation of postoperative scars were also investigated in vivo and in vitro.

## Materials and Methods

### Ethics Statement

The cells used in this study were a primary culture from tenon's capsule tissue (HTFs) obtained from glaucoma filtration surgery patients (n = 7; three females, four males). The cells were cultured at 37°C in 5% CO_2_. Written informed consent was obtained from all patients or their guardians on behalf of the minors enrolled in this study. Ethics approval was obtained from the ethics committee of the Zhongshan School of Medicine, Sun Yat-sen University (Guangzhou, China). The research protocols followed the tenets of the Declaration of Helsinki. The patients' ages ranged from 16 to 56 years with a mean age of 33.39 years (33.39±15.62). Glaucoma was the only ocular disease of all participants, and all a participants had not previously received other ocular surgery or experienced trauma. Fibroblasts that migrated out of the tissue were discarded after passage 5.

All animal studies were strictly performed in compliance with the ARVO Statement for the Use of Animals in Ophthalmic and Vision Research and were approved and monitored by the Institutional Animal Care and Use Committee of Zhongshan Ophthalmic Center (Permit Number: 2011-001). The rabbits used in this study were obtained from the Ophthalmic Animal Laboratory, Zhongshan Ophthalmic Center, Sun Yat-sen University, and all surgery was performed under sodium pentobarbital anesthesia; all efforts were made to minimize suffering.

### Materials

Pluronic F127 (F127) (PEG_99_-PPG_65_-PEG_99_, Sigma-Aldrich) was vacuum dehydrated for 4 h prior to use. The fresh TMC was obtained from Guangdong Huizhou Huayang medical device company and dried overnight in a vacuum oven prior to use. Tetramethylethylenediamine (TMEDA, 98% w/w) was purchased from J & K and used without further purification.

Tetrahydrofuran (THF, A.R), anhydrous ether, ethyl acetate and various other chemicals were purchased from Guangzhou Chemical Reagent Factory. The THF was dried via refluxing over anhydrous calcium chloride and then distilled in the presence of nitrogen protection and sodium. The other chemicals were of reagent grade and used as received.

MMC (Roche Diagnostics GmbH) was dissolved in sterile water (AquaPro; Microgen, West Caldwell, NJ). Dulbecco's modified Eagle's medium (DMEM; Gibco, Grand Island,) supplemented with 10% Fetal Bovine Serum (FBS, Hangzhou Sijiqing Biological Engineering Materials Co, CHN) and Penicillin G-streptomycin sulfate (Life Technologies Corporation. USA) was used as the cell culture medium. The LDH was analyzed using the Cytotoxicity Detection Kit PLUS (Roche Diagnostics GmbH, Germany).

### Synthesis of PTMC_15_-F127-PTMC_15_ copolymer

PTMC_15_-F127-PTMC_15_ was synthesized via the ring-opening polymerization (ROP) of TMC in THF using F127 as an initiator and TMEDA as the catalyst, as previously described [45]. The length of the PTMC segment designed was in view of the solubility of the copolymer in water. The typical experimental procedure is described as follows: TMC (0.3788 g, 3.75×10^−3^ mol) was quickly added to a stirred TMEDA (0.02 ml, 2.5×10^−4^ mol) and F127 (1.575 g, 1.25×10^−4^ mol) in THF (3 ml) in a glove box under an argon atmosphere. The reaction vessel was sealed and placed in an oil-bath thermostat at 55°C. After 48 h of polymerization, the reaction was terminated using two drops of acetic acid. The rough product was isolated via precipitation in cold diethyl ether and dried in vacuo at room temperature to a constant weight.

The ^1^H NMR spectra were recorded in deuterated CDCl_3_ solvent at a concentration of 10 mg/ml on a Mercury-plus 300 MHz at room temperature. Tetramethylsilane (TMS) was used as an internal standard.

The molecular weight and polydispersity were measured using gel permeation chromatography (GPC). The GPC analysis was performed at 40°C using a Waters HPLC 1252 System equipped with a water 2414 RI detector. Tetrahydrofuran was used as the eluent at a flow rate 1.0 ml/min. Monodisperse polystyrene was used as an internal standard.

### Fabrication and properties

#### Sol-Gel transition

The test tube inverting method was used to determine the sol-gel transition temperatures of the copolymer sol in water [46]. Each sample at a given concentration was prepared by dissolving the copolymer in distilled water in a vial and stored at 4°C for 24 h. The vials containing 1 ml copolymer sol were immersed in an oil bath at different setting temperatures and allowed to reach equilibrium. The sample was regarded as a “gel” when flow was no longer visually observed within 30 s by inverting the vial with a temperature increment of 1°C per step.

#### Mass loss of in PBS

One gram sol at a given copolymer concentration (18% w/v for F127 or 5% w/v for PTMC_15_-F127-PTMC_15_) was prepared by dissolving the copolymer in distilled water in a 15 ml vial and stored at 4°C for 24 h. The vials were immersed in a constant temperature oscillation box at 37°C. When sol translated into gel, 10 ml of distilled water was added to the vial. At given time points, the 10 ml of water over the gel was collected and replaced with 10 ml distilled water. The extracted water was vacuum freeze dried to a constant weight, and the mass loss was determined via subtraction. The percentage of mass retained was calculated as follows: the percentage of mass retained (%)  = (*Mt/M_0_*)100, where *Mt* is the mass of stable hydrogel in water at time t and *M_0_* is the original mass of the hydrogel.

#### Drug release in PBS

MMC (Molecular weight, ∼334.33 Da) was dissolved in phosphate buffered saline (PBS, pH 7.4) at a concentration of 1 mg/ml. One-hundred and eighty milligrams of F127 or 50 mg of PTMC_15_-F127-PTMC_15_ were dissolved in an aqueous solution of MMC to obtain 1 g sol. Each sample (300 µl) was placed in 24-well plates in three groups. After gelation, 3 ml of PBS was added to the top of each hydrogel, and the gels were then placed at 37°C. At a predetermined time interval, 100 µl PBS was removed for testing and replaced with 100 µl of fresh media. The experiment was carried out for 16 days. The amount of MMC released from the hydrogel was determined via ultraviolet spectroscopy (Thermo Scientific NanoDrop 2000) at 363 nm. The MMC release was linear between 1 and 20 g/ml (A = 0.02419 +0.08109×C, R^2^ = 0.9995). The cumulative drug release was calculated as follows: Cumulative amount released (%)  =  (*M_t_/M_∞_*) ÿ 100%, where *M_t_* is the amount of MMC released from the hydrogel at time t and *M_∞_* is the amount of MMC loaded in the hydrogel.

### Cell culture

#### Cell immunofluorescence staining

HTFs were fixed in 4% paraformaldehyde in PBS (PFA, pH 7.4) at room temperature for 15 min and permeabilized for 5 min with 0.1% Triton X-100. After being incubated overnight at 4°C with antibodies (mouse Ab against Vimentin (1∶800 dilution) and rabbit Ab against Fibronectin (1∶1000 dilution)), the cells were rinsed and incubated with anti-rabbit secondary antibody (1∶500 dilution) (FITC) and anti-mouse secondary antibody (1∶500 dilution) (Cy3) for 1 hour. Following a series of washes, the slides were treated with 4, 6-diamino-2-phenylindole dihydrochloride(DAPI) for 5 min at room temperature (All antibodies from Sigma–Aldrich). The fluorescence images were observed with a confocal microscope (LSM 510UV-vis Carl Zeiss Company) [47], [48].

#### The cytotoxicity of hydrogel

The cytotoxic effect of PTMC_15_-F127-PTMC_15_ at different concentrations was analyzed at different time points (24, 48 and 72 h) by measuring the lactate dehydrogenase (LDH) leakage using a colorimetric assay. LDH is a stable cytosolic enzyme that is released in the culture medium upon cell lysis. HTF cells were seeded at a density of 1×10^4^ cells/well with DMEM containing 2% (v/v) FBS in a 96-well plate for 24 h according to the manufacturer's instructions [48]. They were then exposed to PTMC_15_-F127-PTMC_15_ hydrogel at different concentrations (3%, 5%, and 10% (w/v)) or to F127 hydrogel (18% (w/v)). At each time point, the supernatant of each well (50 µL) was added to 100 µL of reaction mixture solution for 30 minutes in the dark, followed with 50 µL of stop solution. The microplate was read at an absorbance of 490 nm (Benchmark Microplate Reader; Bio-Rad, Hercules, CA).

Maximally lysed HTF cells were used as the high control group and spontaneous cells were used as the low control group. The amount of cell toxicity was directly proportional to the LDH activity and calculated as follows: LDH release (%)  =  [OD of (Experimental – Low control)/OD of (High control – Low control)] ×100%.

#### The cell apoptosis induced by hydrogel

An annexin V-FITC apoptosis detection kit (BD Pharmingen, USA) was used to detect apoptotic cells according to the manufacturer's protocol and quantified using Fluorescence Activated Cell Sorting (FACS). Annexin V binds to the plasma membrane of apoptotic cells, and PI stains the DNA of cells with compromised cell membranes [47]. The cells were seeded into a 35 mm plate at 1×10^6^ cells/well for 24 h at 37°C and then treated with PTMC_15_-F127-PTMC_15_ (5% w/v) or F127 (18% w/v). Untreated cells were used as a blank control. After being incubated for 12, 24, 48 and 72 h, both monolayer cells and those in the supernatant were collected by trypsinization and centrifugation at 1000 g for 5 min. After being washed three times with PBS, the samples were then re-suspended in 100 µL binding buffer, followed by incubation with 5 µL Annexin V-FITC and 5 µL PI in the dark at room temperature for 15 minutes. Thereafter, the stained cells were analyzed using a flow cytometer (FACSCalibur, Becton Dickinson, USA). The data were analyzed using the CellQuest software (Becton Dickinson, USA).

#### The cytotoxicity of MMC-loaded hydrogel

According to the manufacturer's instructions mentioned above, the cytotoxicity of MMC was analyzed by detecting the LDH released by MMC-treated HTF cells into the culture medium. In brief, 50 µL of conditioned medium alone were sampled from 96-well plates containing 1×10^4^ HTF cells/well after incubation with different concentration of MMC (0, 25, 50, 100, 200, 400 µg/ml) for 24 and 48 hours.

The toxicity of the MMC-loaded hydrogel was also tested using the LDH release assay. The supernatant cultures were obtained from four treatment groups after 24, 48, and 72 h to assess the LDH release: HTF cells were incubated with 0.1 ml of PTMC_15_-F127-PTMC_15_ hydrogel (5% w/v) loaded with 0.1 mg/ml MMC, 0.1 ml F127 hydrogel (18% w/v) loaded with 0.1 mg/ml MMC, 0.1 mg/ml MMC only or with 0.5 mg/ml MMC for 5 minutes. Untreated cells served as the control group.

#### The cell apoptosis induced by MMC-loaded hydrogel

Apoptosis was detected in the five groups using Annexin V-FITC staining and FACS as described above after 12, 24, 48 and 72 h. The data were also analyzed using the CellQuest software (Becton Dickinson, USA).

### Animal Studies

Forty New Zealand white rabbits, aged 10 to 14 weeks and weighing 2.0 to 2.5 kg, were used in this study. The rabbits were randomly divided into four different treatment groups of ten rabbits each, as summarized in table 1. The surgical eye was randomly determined. The observers were blinded to all group information.

**Table 1:** Random divided animal groups.

Groups	n	Treatment in the filtration surgery
a (blank control)	10	Undergoing the filtration surgery with Balance Saline Solution(BSS).
b	10	0.1ml hydrogel of 5% PTMC_15_-F127-PTMC_15_ loaded with 0.1 mg/ml MMC was injected during filtration surgery.
c (positive control)	10	A sponge (3 mm×4 mm) soaked with MMC solution (500 µg/mL) was placed under the conjunctival flap intraoperatively. After 5 min, it was removed and all the exposed tissues were irrigated with 100 mL BSS.
d (negative control)	10	0.1ml hydrogel of 5% PTMC_15_-F127-PTMC_15_ with no drug loaded was injected in filtration surgery.

Prior to the filtration surgery, the hydrogel was prepared by dissolving the polymer using sterile water with or without 0.1 mg/ml MMC. The hydrogel was then stored at 4°C for gelation. The surgeries were performed under a surgical microscope (Zeiss, Germany).

#### Filtration surgery in rabbits

Filtration surgery was performed based on a standard protocol by the same surgeon. The rabbits were anesthetized by an intramuscular injection of ketamine hydrochloride (50 mg/kg) plus chlorpromazine hydrochloride (25 mg/kg) (both from Gutian Pharmaceutical Company, China) prior to surgery. Topical anesthesia was induced with proxymetacaine hydrochloride drops (Alcaine 0.5%). Under surgical asepsis, a limbal-based conjunctival incision was designed, followed by a partial-thickness 4×4 mm rectangular scleral flap. A 2×2 mm sclerectomy and a peripheral iridectomy were then performed. The scleral flap was repositioned and closed with two interrupted 10–0 monofilament nylon sutures (Alcon, USA). The whole tenon's capsule was closed with interrupted sutures. The conjunctival incision was closed with a running 8–0 absorbable polyglactin suture to yield a watertight closure. MMC (0.5 mg/ml×0.1 ml) was applied for 5 min prior to the sclerectomy in the MMC-5 min group.5% PTMC_15_-F127-PTMC_15_ (0.1 ml, with or without 0.1 mg/ml MMC) was injected into the subconjunctival space with a 30G cannula before the conjunctival incision was completely closed.

#### Clinical observation

The clinical examinations were assessed before and after surgery (days 1, 3, 5, 7, 10, 14, 28). Slit lamp examinations were performed to inspect the anterior chamber depth, surgery complications, such as corneal edema, intraocular inflammation or hemorrhage, and bleb conditions. The intraocular pressure (IOP) was measured in both eyes with an applanation tonometer (Tono-Pen Avia; Reichert, Inc., Depew, NY) under topical anesthesia. Bleb survival was evaluated to indicate the efficacy of the surgery. According to the bleb size measurement method described by Cordeiro et al. [49], the bleb width and depth was calculated with caliper measurements and the height was semi-quantitatively graded using slit-lamp examinations (0, flat; 1, shallow/formed <1 mm; 2, elevated <2 mm; 3, high >2 mm). Bleb failure was defined as the appearance of a flat, vascularized and scarred bleb associated with a deep anterior chamber.

#### *In vivo* toxicity evaluation

To assess the in vivo toxicity, the corneal endothelial cell density was examined before and 28 days after surgery. The MMC concentrations of the aqueous anterior chamber in group b and c were also tested at the Guangzhou Analysis and Testing Center using liquid chromatograph-mass spectrometer (LC-MS), which has a minimum detectable concentration of 0.5 ng/ml. Two rabbits were randomly selected from each group 3, 7 and 14 days after surgery. Topical anesthesia was applied at each predetermined time, and 0.1 ml aqueous humor was extracted from the corneal limbus using a 1-ml syringe with a 30 G needle (BD, La Jolla, CA) under a surgical microscope. The samples were then immediately preserved on ice until being analyzed within 1 hour.

#### Histological examination

After 28 days, the rabbits (four in each group) were killed via a lethal intravenous injection of excess phenobarbitone, and the eyeballs were enucleated and prepared for histological examination.

All eyes were immersed in a formalin acetic acid (FAA) alcohol solution for no less than 24 hours, and then stored in 70% alcohol and fixed in paraffin wax. Sequential 5 µm sections of the operative region were prepared and stained with hematoxylin and eosin (H&E) to obtain a general impression of total cellularity, with Massones Trichrome and α-SMA antibody to identify collagen fibers deposition. To reveal the proliferation activity in the wound site, the proliferating cell nuclear antigen (PCNA) immunohistochemistry was tested using PCNA primary antibody (1∶800; Millipore, Boston, MA). The terminal deoxynucleotidyl transferase-mediated dUTP nick end labeling (TUNEL) method was used (In Situ Cell Death Detection) to analyze the apoptotic cells around the surgical wounds (In Situ Cell Death Detection Kit POD, Roche Applied Sciences, Indianapolis). The numbers of positively stained cells were counted in five high-power fields for statistical analysis.

### Statistical Analysis

All data are expressed as the mean±standard deviation (SD). An analysis of variance (ANOVA) was used to test the differences among the groups. A Bonferroni post hoc test was then used to determine significant differences between pairs of relevant groups. P<0.05 was considered statistically significant.

## Results

### Characteristic of PTMC_15_-F127-PTMC_15_ block copolymer

**Figure 1:**
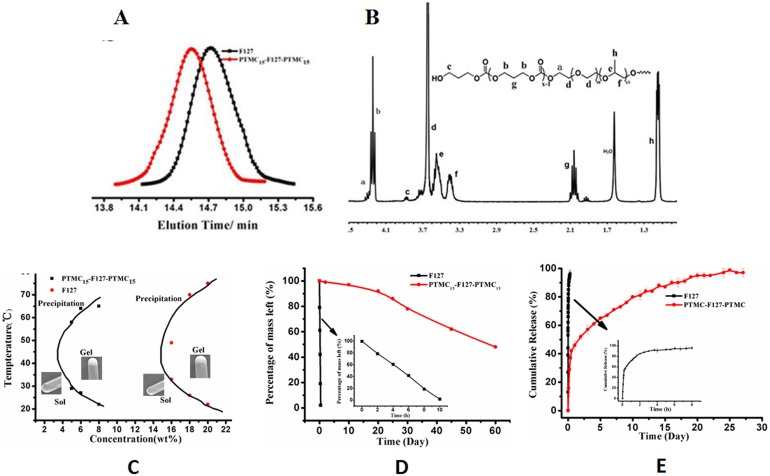
Characterization and the properties of the hydrogel. (A) GPC analysis (in THF at 40°C); (B) ^1^H NMR spectrum of the block copolymer (in CDCl_3_); (C) Phase diagrams by invert test; (D) mass lose profiles; E, MMC release kinetics of/from PTMC_15_-F127-PTMC_15_ hydrogel (5% w/v) and F127 hydrogel (18% w/v) in PBS (pH = 7.4) at 37°C, MMC (1 mg/ml).

The ring-opening polymerization of TMC was initiated by F127 and catalyzed by TMEDA at 55°C for 48 h. After finishing the reaction, gel permeation chromatography (GPC) was used to confirm the product. The GPC curve (Figure 1A) of F127 disappeared, and the elution curve appeared in a short time at approximately 1.3×10^4^ g/mol in a monomodal manner (PDI = 1.27), suggesting that the polymerization of TMC was successful.

In addition to the characteristic resonance peaks of F127 at 1.15 ppm [h] and 3.40–3.75 ppm [f, e, d], new resonance signals indicative of the methylene protons of the TMC segment appeared at 4.25 ppm [b] and 2.05 ppm [g], as shown in Figure 1B. The length of PTMC incorporated into the two ends of F127 can be calculated from 195*I*_g_/2*I*_h_ (*I*_h_ is integration of -CCHC***H***_3_C- in PPG ÿ1.15 ppm); *I*_g_ is the integration of -CH_2_C***H***_2_CH_2_- in PTMC ÿ2.05 ppm), actual value was 15). Therefore, a well-defined PTMC_15_-F127-PTMC_15_ block copolymer was obtained.

### Properties of temperature-responsive PTMC_15_-F127-PTMC_15_

As shown in Figure 1C, the incorporation of the hydrophobic PTMC segments into the two ends of the F127 molecules decreased the critical gelation concentration (CGC) from 15% (w/v) to 3.5% (w/v). Moreover, the sol-gel transition temperature at the tested concentrations was between 20–30°C, which is much lower than normal body temperature. The degradation assay (Figure 1D) showed that the F127 hydrogel rapidly lost mass and disappeared in the aqueous medium within 8 hours, even at a concentration of 18% w/v in PBS buffer solution (pH 7.4, 37°C). On the contrary, the PTMC_15_-F127-PTMC_15_ (5% w/v) hydrogel exhibited slow mass loss. Obvious degradation was not observed in the first 10 days, and less than 14% mass loss was found after 16 days in this copolymer. Moreover, the stability of the hydrogel could be improved by increasing the concentration of copolymer solution; for example, the shape was maintained for 40 days for 6% w/v copolymer hydrogel and 60 days for 7% w/v hydrogel.

After the formation of the MMC-loaded hydrogel, the drug release properties were investigated at 37°C. The corresponding release profiles are exhibited in Figure 1E. Approximately 57% of the incorporated MMC was released from the PTMC-modified F127 hydrogel in the first 24 h. The cumulative drug release increased gradually to 94% in the following 16 day. Conversely, approximately 65% of the MMC incorporated in the F127 hydrogel was released in the first 20 min, and another 30% was released in the following 100 min. The residual 5% of MMC was gradually released over 8 h.

### The cytotoxicity and the cell apoptosis incubated with hydrogels

**Figure 2:**
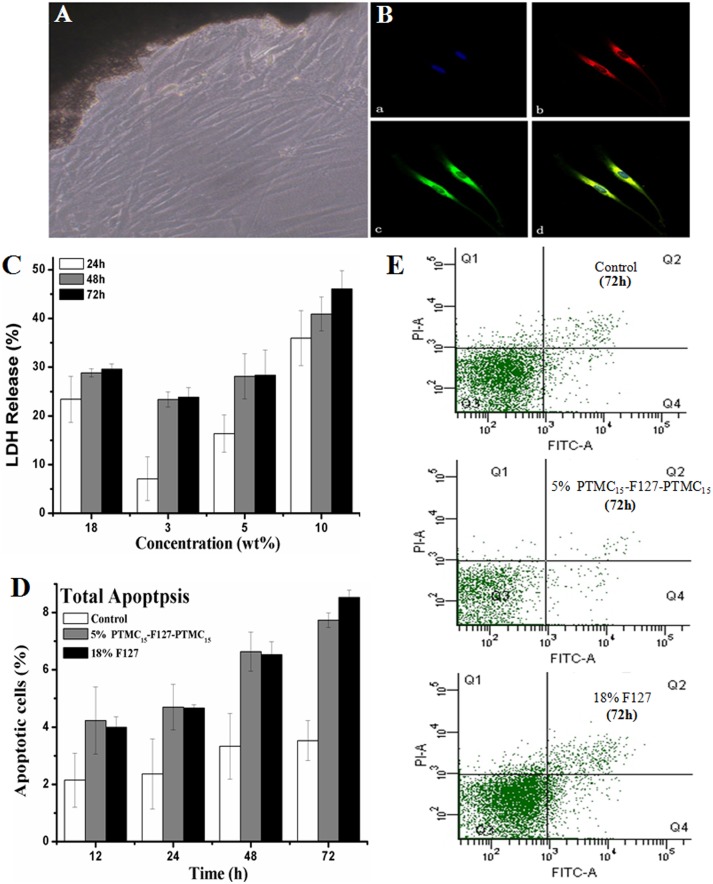
In vitro cell toxicity of the HTFs cocultured with PTMC_15_-F127-PTMC_15_ copolymer in different concentration and with F127. (A) primary cultured HTFs were identified with microscopy and (B) with immunofluorescence. (a) Negative control using DAPI dyed the nucleus (blue) of HTFs. (b) Vimentin antibody (red) was added. to primary HTFs (c) Fibronectin antibody (green) was added. (d) Melt. (C) LDH release assay were used to analyze cell toxicity in different concentration of PTMC_15_-F127-PTMC_15_ (3%, 5% and 10% w/v) and in 18% w/v F127 within 3 days. (D) Total apoptosis of HTFs incubated with 0.1 ml 5% w/v PTMC_15_-F127-PTMC_15_ or 0.1 ml 18% w/v F127 were detected by Annexin V/PI staining using fluorescence-activated cell sorting (FACS). (E) Apoptotic changes between 5% w/v PTMC_15_-F127-PTMC_15_ and 18% w/v F127 at 3 days. LDH  =  lactate dehydrogenase. ‘Control’ one is the group with no treatment.

The outgrowth of primary cultured cells from the edges of human Tenon's capsular tissues could be obtained within 1 week (Figure 2A), and culture confluency was reached within 2–3 weeks. The cells were identified via the positive staining of fibroblast-specific molecular markers and the cytoskeletal molecules vimentin and fibronectin (Figure 2B).

By testing the LDH release, which has widely been used as a reliable marker of cellular injury, from HTFs with different concentrations of PTMC_15_-F127-PTMC_15_ and F127, we found that the cytotoxicity was dose- and time-dependent (Figure 2C). The LDH release rate positively correlated with the copolymer concentration of PTMC_15_-F127-PTMC_15_ at 3%, 5% and 10% w/v (1 d: 7%, 16.4%, 36%, 2 d: 16.4%, 28.1%, 28.8%, respectively) and significantly increased with the incubation time up to 3 d (3%: 24%, 5%: 28.3% and 10%: 46.1%). In view of the CGC of the copolymers in aqueous solution, 5% (w/v) PTMC modified hydrogel from polyether-ester was selected as the practical concentration in the application, and the cytotoxicity was lower than that of the 18% (w/v) F127 hydrogel (1 d: 16.4% vs. 23.4%, 2 d: 28.1% vs. 28.8%, and 3 d: 28.3% vs. 30%).

Moreover, the FACS analysis of cells apoptosis induced by 18% F127 and 5% PTMC_15_-F127-PTMC_15_ (Figure 2D–2E) revealed that the percentage of apoptotic cells was a function of time, even though statistically significant differences were not observed between the two groups within three days (12 h: 4.2% vs. 4%, 1 d: 4.7% vs.4.7%, 2 d: 6.6% vs. 6.5%, 3 d: 7.7% vs. 8.5%. Bonferroni test, p72 h = 0.418).

### Evaluation of PTMC_15_-F127-PTMC_15_/MMC delivery system *in vitro*

#### Toxicity Assessment of MMC

**Figure 3:**
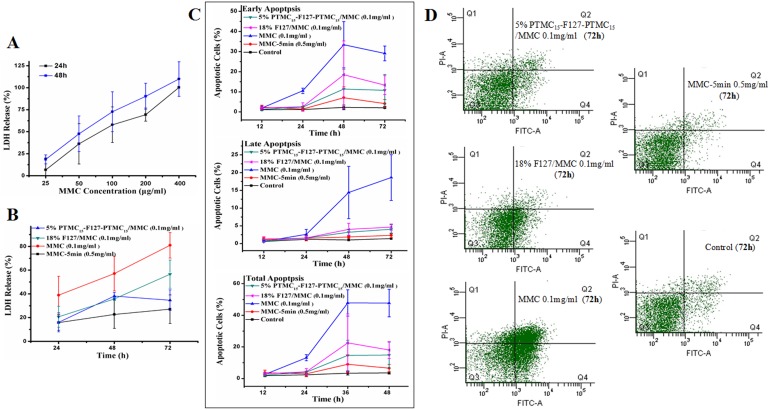
In vitro toxicity of HTFs incubated with MMC. (A) LDH release from cells incubated with different concentrations of MMC within 48 hours. (B) Cell viability of HTFs treated with 0.1 mg/ml MMC loaded 5% w/v PTMC_15_-F127-PTMC_15_ or 18% w/v F127 were measured by LDH assay for 3 days. (C) Early stage, Late stage, and Total HTFs apoptotic cells change labeled by Annexin V/PI in different treatment groups were detected by FACS for 3 days. (D) The FACS for Annexin V/PI staining of HTFs treated with 0.5 mg/ml MMC for 5 minutes, with two type of hydrogel loaded with 0.1 mg/ml MMC and 0.1 mg/ml MMC at 3 days. LDH  =  lactate dehydrogenase. ‘Control’ group is the group with no treatment.

As shown in Figure 3A, MMC is strongly cytotoxic. The cell viability negatively correlated with the concentrations in MMC from 0 to 400 µg/ml. The cytotoxicity rate in the first 24 h was 6.6% at 25 µg/ml MMC and reached 90% at 400 µg/ml, which suggested that high doses of MMC induced cell death. Moreover, the cell toxicity significantly increased over time at one concentration; even at a modest concentration of 25 µg/ml, the cell toxicity increased from 6.6% to 18.6% after 48 h.

#### Efficacy evaluation

Figure 3B illustrates that cells co-cultured with 0.1 ml F127 hydrogel (18% w/v) loaded with 0.1 mg/ml MMC induced significantly more cell injury than the 5-min 0.5 mg/ml MMC group did in 72 h (50.6% versus 27.1%, Bonferroni test p = 0.014) because all of the MMC was released from the F127, which dissolved in 1 day. The cell toxicity of 0.1 ml PTMC modified F127 hydrogel (5% w/v) loaded with 0.1 mg/ml MMC was similar to that of the MMC-5 min group over 3 days (1 day: 16.0% vs. 15.8%, 2 day: 38.1% vs. 22.7%, 3 day: 34.7% vs. 27.1%, Bonferroni test p>0.05). Additionally, the 0.1 mg/ml MMC-treated group has the strongest cytotoxicity for the maximum amount MMC it was treated with.

In addition, we performed Annexin-V/PI staining and sorted the cells using FACS (Figure 3C). The apoptosis rate of the MMC group was significantly higher during the first 12 h (2.36% in early stage and 1.3% in late stage) due to its high initial concentration. However, this rate decreased to a minimum over time. Although the differences were not statistically significant, the 18% F127 sustained group induced a higher cell apoptosis rate than the 5% PTMC modified group did in 72 h (14.8% vs. 18%). Moreover, compared with the MMC-5 min group at 72 h (Figure 3D), the 18% F127 sustained group induced more cell apoptosis in the early stage (13.3% vs. 4.2%, p = 0.017) and overall (18% vs. 6.5%, p = 0.027), but no difference was found in the PTMC modified F127 sustained group (p>0.05). Cells treated with 0.1 mg/ml MMC showed a higher apoptosis rate than any of the other groups after 24 h (p = 0).

### Evaluation of PTMC_15_-F127-PTMC_15_/MMC delivery system *in vivo*

All animals survived with no remarkable differences. No case of filtering bleb leakage and other complications were observed.

#### Filtering bleb survival Analyze

**Figure 4:**
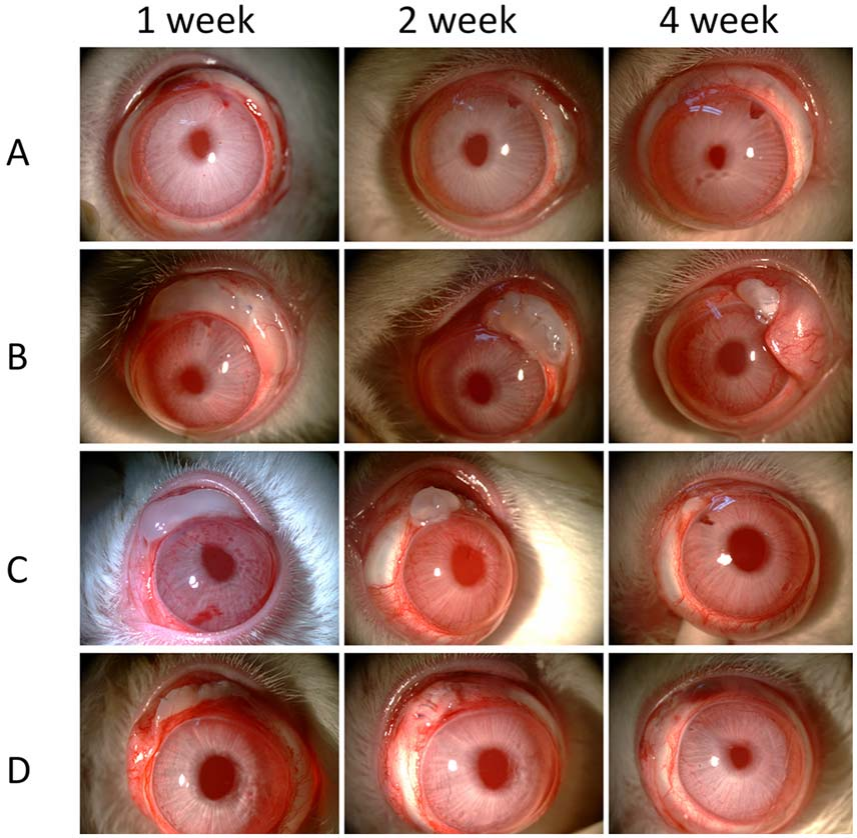
Postoperative observation in rabbit glaucoma filtration surgery models. (A) Group had filtration surgery only. (B) Group injected with 0.1 ml 5% w/v PTMC_15_-F127-PTMC_15_/MMC (0.1 mg/ml) hydrogel in glaucoma filtration surgery. (C) Treat with 0.5 mg/ml MMC for 5 minutes during glaucoma filtration surgery group. (D) Group with 0.1 ml 5% w/v PTMC_15_-F127-PTMC_15_ injected in glaucoma filtration surgery.

The filtering bleb characteristic is the most important indicator of a successful operation. Diffused filtering blebs appeared in four groups 3 days after surgery. Blebs failed within 7 days after surgery in groups a and d. The inflated blebs in group c began to shrink and thinned, showing avascular changes within 20 days, while the filtering blebs remained flat on day 28 in group b. Figure 4 depicts the typical appearance of blebs in the 4 weeks after surgery.

**Figure 5:**
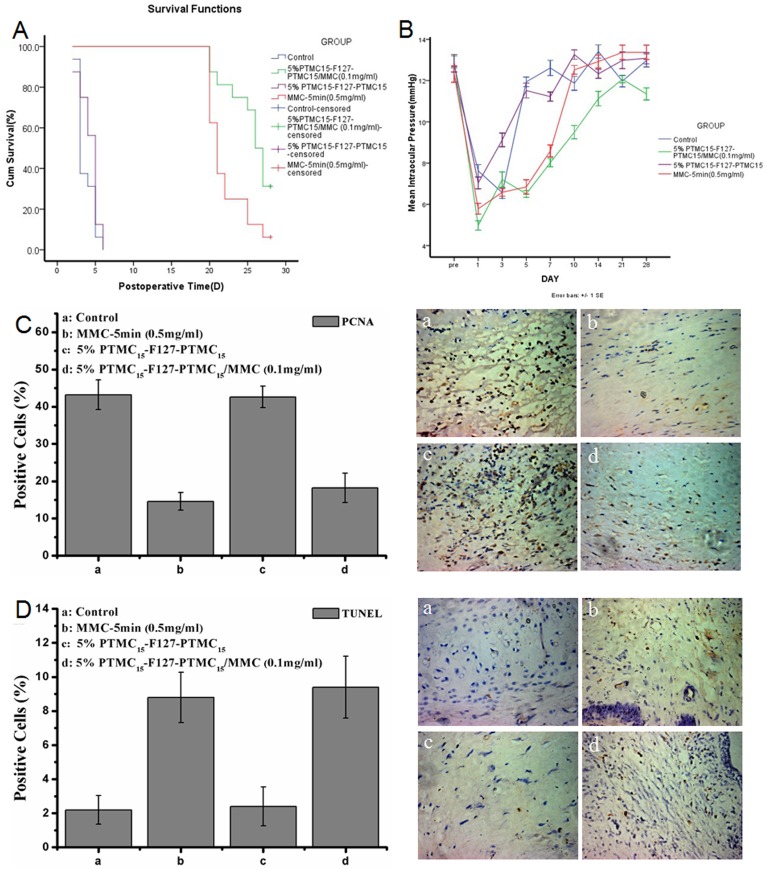
Postoperative evaluations in rabbit models. The rabbits were divided into four treatment groups: ‘Control group’ is the filtration surgery only group (a). Group injected with 0.1 ml 5% w/v PTMC_15_-F127-PTMC_15_/MMC (0.1 mg/ml) hydrogel in filtration surgery (b). Treat with 0.5 mg/ml MMC for 5 minutes during filtration surgery group (c). Group with 0.1 ml 5% w/v PTMC_15_-F127-PTMC_15_ injected in filtration surgery (d). (A) Survival curve of filtering blebs after glaucoma filtration surgery using Kaplan-Meier analysis. Filtering blebs in group ‘a’ (n = 4) and ‘d’ (n = 4) mostly failed within 1 weeks. Group ‘b’ (n = 4) markedly increased the bleb survival period. In group ‘c’ (n = 4), the bleb survived nearly 4 weeks. Kaplan-Meier analysis showed a significant difference in the survival distributions among the four groups. (Log Rank  = 75.121, p<0.01). (B) IOP changes within postoperatively 28 days. The results were obtained and expressed as the mean± SD. There is no significant difference between each group (p>0.05). (C) Proliferating cell nuclear antigen (PCNA) was analyzed in each group at post-operative day 28.(D) Apoptotic cells were indicated by TUNEL assay in each group at day 28.

As shown in Figure 5A, a Kaplan–Meier survival curve analysis indicated that the bleb survival in the MMC treatment groups was significantly prolonged (log rank  = 75.121 p = 0). The mean bleb survival times 3.7±1.1, 25.5±2.9, 22.1±2.7 and 4.3±1.3 days in group a, b, c and d.

#### Intraocular pressure (IOP) Examination

Figure 5B illustrates that the IOPs differed among the four groups. For each group, the IOP reduced during the early stage after operation and increased slowly over time. After using repeated measurement, we found that the IOP in group b and c has drop more than other groups (Bonferroni test, p<0.001), although no significant difference was found between them. Meanwhile, IOP in four groups were back to baseline at day 21, and significant differences were not found at 28 days among groups (p>0.05).

#### Toxicity Tests in anterior chamber

LC-MS did not detect MMC in the aqueous humor in groups b and c 3, 7 and 14 days after surgery.

Table 2 shows no significant corneal endothelial cell count changes in groups with or without PTMC15-F127-PTMC15 application. (p>0.05).

**Table 2:** Corneal Endothelial Cell Count (/mm^2^, mean±SD).

Groups	n	pre-operative	28 days post-operative	p value
Control	4	2272.2±172.5	2295.7±178.6	0.594
PTMC_15_-F127-PTMC_15_/MMC	4	2385.4±153.0	2366.6±174.1	0.057
MMC-5min	4	2307.2±169.7	2334.1±174.6	0.81
PTMC_15_-F127-PTMC_15_	4	2272.6±180.2	2278.4±171.2	0.723
p value		0.145	0.195	

#### Histological Features

**Figure 6:**
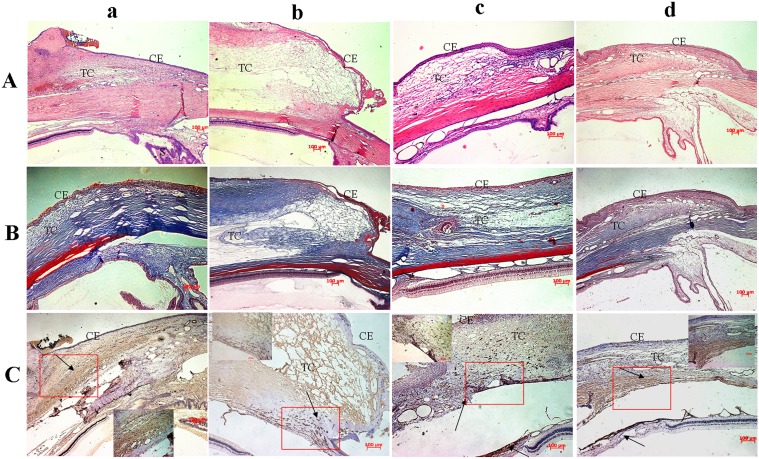
Postoperative Histological characteristics evaluations in rabbit models. The rabbits were divided into four treatment groups: Filtration surgery only group (a). Group injected with 0.1 ml 5% w/v PTMC_15_-F127-PTMC_15_/MMC (0.1 mg/ml) hydrogel in filtration surgery (b). Treat with 0.5 mg/ml MMC for 5 minutes during filtration surgery group (c). Group with 0.1 ml 5% w/v PTMC_15_-F127-PTMC_15_ injected in filtration surgery (d). (A) In H&E staining, the subconjunctival scarring in group ‘a’ were noticed with fibrocellular scar tissue and with closed sclerotomy, group ‘b’ had loosely arranged subepithelial connective tissues, group ‘c’ had a few inflammatory cells and thin subconjunctival tissue, and group ‘d’ was similar to group ‘a’. (B) Massones Trichrome staining showed group ‘a’ and ‘d’ had more dense collagen tissues in sub-conjunctiva space than group ‘b’ and ‘c’. (C) Immunohistochemical examination of α-SMA illustrated that lesser α-SMA specific myofibroblast were differentiated in group ‘b’ and ‘c’. CE: conjunctival epithelium; TC: Tenon's Capsule;

Figure 6(A–B) presents the histological sections of the subconjunctival tissue around the surgical area in the four groups 28 days after surgery. In group a, the subconjunctiva filtration space had disappeared, the sclerotomy was closed and massive fibrotic scar tissue and dense collagen tissue formed. Groups b and c showed a mild fibrotic response and collagen deposition with loosely arranged sub-epithelial connective tissue in the filtration area. The drainage channel also closed in group d, with numerous activated fibroblasts and a large amount of dense collagen tissue was evident.

The immunochemistry examination of α–SMA in the filtration space shows a marked increase in the α-SMA-expressing myofibroblasts in groups a and d compared to groups b and c.(Figure 6C).

The PCNA results are shown in Figure 5C. The rate of division of positive cells was significant higher in groups a and d (43.2±4 and 42.6±3) than groups b and c (18.2±4 and 14.6±2, respectively, p>0.05).

Figure 5D shows the TUNEL results. The number of apoptotic cells significantly increased in groups b and c (9.4±1.8 and 8.8±1.5) compared to the other groups (A = 2.2±0.8, D = 2.4±1.1; n = 4, p<0.01).

## Discussion

In contrast to the other antiproliferative agents [13], [50]–[59], MMC, which can effectively inhibit fibroblast cell migration and extracellular matrix production, is the most frequently employed single-use adjunct in glaucoma surgery. A survey of members of the American Glaucoma Society from 2002 showed that more than 68% of surgeons used MMC in glaucoma filtration surgery cases [9]. However, high failure rates persist, especially in surgeries with complicated glaucoma, such as patients with pediatric glaucoma, which show a success rate of only 59% over a 24-month interval [60]. Moreover, MMC shows strong cytotoxicity, and the toxic effect is enhanced by increasing the drug concentration, as we demonstrated in this study.

Unlike the uncontrollable drug bounding amount and unstable drug release rate in some biomaterials [47], [61]–[65], the in situ forming thermo-sensitive hydrogel fulfilled many advantages required for glaucoma filtration surgery. One of the great benefits is that the drug can be incorporated during the formation of the hydrogel, which allows the precise control of the concentrations of incorporated drugs [66]. Furthermore, its simple preparation can avoid the denaturation of bioactive proteins and peptides that results at extreme conditions (e.g. heating, organic solvent, etc.) [67]. In addition, it can sustain release of both hydrophilic and hydrophobic drugs; thus, the drug content in the hydrogel is only limited by the drug solubility [68], [69].

Our in situ forming thermo-sensitive injectable hydrogel PTMC_15_-F127-PTMC_15_ was developed with several additional PTMC moieties on the two ends. This new hydrogel has a low viscosity, good water-solubility to a certain degree of polymerization, which was easily modulated by the feed ratio, as shown in Figure 1. Different from most other synthetic injectable hydrogels, which feature a relatively high working concentration (e.g., PLGA-PEG-PLGA for 20% w/v [70], F127 for 18–35% w/v [67], [71]), this hydrogel allows for a wider sol-gel window (Figure 1C) at lower concentration (5% w/v).

Moreover, PTMC_15_-F127-PTMC_15_ is highly biocompatible. After modification with the oligo-hydrophobic polymer PTMC (PDI = 30), the PTMC_15_-F127-PTMC_15_ showed good water solubility at 4°C and formed a gel at 37°C. This material was no more cytotoxic than 18% F127 according to in vitro LDH and Annexin V/PI tests. We also confirmed this finding in rabbit filtration surgery models. No obvious inflammation, tissue necrosis, cornea endothelium cell loss or other adverse reactions were observed in the clinical and histological sections 28 days after surgery. The results imply that this novel hydrogel has potential as a drug carrier for glaucoma filtration surgery.

Beyond that, PTMC_15_-F127-PTMC_15_ is a promising biomaterial carrier for the sustained release of MMC after glaucoma surgery which can enhance the stability (Figure 1D), prolong the drug release time (Figure 1E) and reduce the cytoxicity of MMC in vivo and in vitro. As the in vitro release study showed, the release of MMC can be sustained for more than 2 weeks in our drug release system after an initial burst release of approximately 57% during the first day. Moreover, compared with the MMC-5 min group, the in vitro toxicity results showed that neither the initial MMC burst during the first 12 h nor the total release observed during the three observation days showed more cell apoptosis in the sustained release group. In contrast, the 18% F127 containing MMC resulted in significantly more cell injury due to the degradation of 18% F127 within 10 hours. The in vivo aqueous humor test and cornea endothelium cell count also revealed that none of the MMC entered the anterior chamber in the sustained release group.

In addition, the MMC sustained release group was more effective than a 5-minute application of 0.5 mg/ml MMC in the rabbit model (Figure 4). The filtering bleb survival and histological analysis rather than IOP were used as the primary outcome measurement in the current normotensive IOP model studies [72]–[76]. Although the number of days that blebs persisted did not differ, our data indicated that the blebs in the PTMC_15_-F127-PTMC_15_/MMC treatment group were larger than those in the MMC-5 min group (Figure 5B) 28 days after surgery, indicating that the anti-scarring effects of the drug loaded-hydrogel were better and the infiltration area was larger. The bleb survival in the MMC sustained group may have extended beyond the observation period of 28 days. Moreover, the sustained release group showed less fiber formation and a looser deposited collagen structure in the subconjunctival spaces than the MMC-5 min group according to HE and Masson trichrome staining. Furthermore, the specific marker for the myofibroblast cell activation of α-SMA and the proliferation cell antibody PCNA indicated a reduction in fibroblast cell activation in the sustained release group, although the difference between the sustained release group and the MM-5 min group was not significant. These data indicate that using PTMC_15_-F127-PTMC_15_ as a drug vehicle for the controlled release MMC helps to regulate postoperative scarring. By promoting bleb survival, maintaining IOP and reducing complications, this novel hydrogel-MMC system might benefit glaucoma filtration surgery, especially for those patients with a high risk for surgery failure.

## Conclusion

Using ^1^H NMR and GPC characterization, we have confirmed that a novel thermo-sensitive PTMC modified F127 hydrogel was synthesized with the advantages of a low work concentration, stable mechanical properties, appropriate MMC release profile and good compatibility. Ultraviolet spectroscopy measurements show that MMC was successfully loaded into this PTMC15-F127-PTMC15 hydrogel and that the full drug release time reached 25 days. Moreover, the in vitro LDH release and FACS analysis in conjunction with the in vivo clinical and histological examination suggest that PTMC15-F127-PTMC15 showed good compatibility with low toxicity. Furthermore, the in vitro HTFs and in vitro rabbit filtration surgery models indicate that PTMC15-F127-PTMC15 loaded with MMC functions more effectively. In brief, our data demonstrated that the PTMC15-F127-PTMC15 hydrogel is a stable release, effective and safe MMC carrier and can be considered a practical new drug delivery system using in glaucoma surgery.
